# Long-Term Survival in Canine Hepatosplenic T-Cell Lymphoma Treated with Toceranib Phosphate Following Splenectomy: A Case of Atypical Lymphoma

**DOI:** 10.3390/vetsci11100458

**Published:** 2024-10-01

**Authors:** Makoto Akiyoshi, Masaharu Hisasue, Midori Goto Asakawa, Sakurako Neo

**Affiliations:** 1 Laboratory of Small Animal Internal Medicine, School of Veterinary Medicine, Azabu University, Sagamihara 252-5201, Kanagawa, Japan; akiyoshi@azabu-u.ac.jp; 2 Akiyoshi Animal Clinic, Yamato 242-0008, Kanagawa, Japan; 3 Clinical and Anatomic Pathology Unit, Veterinary Specialists Emergency Center, Kawaguchi 333-0823, Saitama, Japan; mi-asakawa@vsec.jp; 4 Laboratory of Clinical Diagnosis, Azabu University, Sagamihara 252-5201, Kanagawa, Japan; neo@azabu-u.ac.jp

**Keywords:** dog, GL lymphoma, hepatic lymphoma, hepatosplenic lymphoma, LGL lymphoma, toceranib

## Abstract

**Simple Summary:**

This study focused on a 10-year-old dog presenting with weight loss, excessive drinking, and liver-related issues. The aim was to understand a rare type of lymphoma affecting the liver and spleen, known as hepatosplenic T-cell lymphoma. By examining tissue samples from the spleen, liver, and gallbladder, researchers identified abnormal cells indicating hepatosplenic T-cell lymphoma. Surgery and subsequent treatment with toceranib, a drug not commonly used for this lymphoma in dogs, led to a notable improvement in the dog’s condition. This finding suggests toceranib could be an effective treatment option for hepatosplenic T-cell lymphoma in canines. The study’s results highlight the potential of new treatments for rare diseases in pets, offering hope for improved care and outcomes in veterinary oncology.

**Abstract:**

Toceranib phosphate (toceranib) is approved for canine mast cell tumor treatment. However, no long-term response to toceranib in canine HSTCL has been reported. Here, we describe a case of a 10-year-old castrated mixed-breed dog that presented with a 3-month history of weight loss, polydipsia, and polyuria. The clinicopathological and imaging abnormalities included icterus, biliary obstruction, and splenomegaly with multiple diffuse splenic hypoechoic nodules. On day 21, a cholecystectomy was performed to remove the obstruction, followed by a liver biopsy and splenectomy. Cytology of the spleen and liver showed many small lymphocytes with intracytoplasmic granules (sGLs). Splenic and hepatic infiltration of neoplastic CD3/granzyme B-positive small cells and lymphocytic cholecystitis with granzyme B-negative small cells were noted. T-cell receptor gene clonal rearrangements were observed in the liver tissues. The dog was diagnosed with a hepatosplenic T-cell lymphoma (HSTCL) of sGLs concurrent with lymphocytic cholecystitis. The icterus resolved after surgery, but there was progressive elevation of liver enzyme levels. Toceranib was administered from day 39, resulting in decreased liver enzyme levels, and the dog remained in good condition. The dog stayed in remission after toceranib administration and survived for 460 days. Toceranib should be considered an effective treatment option for canine HSTCL.

## 1. Introduction

Canine hepatosplenic T-cell lymphoma (HSTCL) is a lymphoma centered in the liver and spleen without significant involvement of peripheral lymph nodes and is thought to arise from cytotoxic ɤδ T-cells in the spleen [1,2]. Cytologically, canine HSTCL is characterized by intermediate to large lymphocytes, with most cases reported as granular lymphocytes (GLs), lymphomas containing azurophilic granules in the cytoplasm. Thus, canine HSTCL cases are not reported to have predominantly small-sized GLs (sGLs) [1,2,3,4]. Canine HSTCL is a relatively rare subtype of lymphoma with poor prognosis and limited response to chemotherapeutic agents, such as the CHOP-based protocol (CHOP: vincristine, cyclophosphamide, doxorubicin, and prednisolone), lomustine (CCNU), nimustine (ACNU), and L-asparaginase, which are used in many canine lymphomas [1,2,3,4]. To date, no effective chemotherapeutic agents have been reported against HSTCL.

Toceranib phosphate (toceranib) is a tyrosine kinase inhibitor that targets platelet-derived growth factor receptors (PDGFRs), vascular endothelial growth factor receptors (VEGFRs), and KIT [5]. It has been approved for the treatment of canine mast cell tumors, and some studies have reported its efficacy in dogs with several types of carcinomas [6,7,8]. However, no long-term response to toceranib in canine HSTCL has been reported. Herein, we describe a case of canine HSTCL with sGLs treated using toceranib after a splenectomy.

## 2. Case Presentation

**Figure 1:**
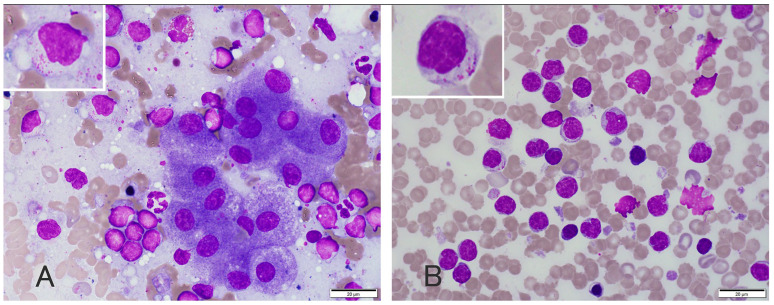
Photomicrographs of liver (**A**) and spleen (**B**) cytology, and Wright and Giemsa staining. Cytological analysis of the liver and spleen revealed a moderate number of small lymphocytes with clusters of well-differentiated hepatocytes. The lymphocytes had small (up to 1.5 × RBC) nuclei with indistinct nucleoli and small amounts of light blue cytoplasm with small amounts of fine, red to magenta granules.

A 10-year-old, 19 kg, castrated male mixed-breed dog was presented with a 3-month history of weight loss, polydipsia, and polyuria. Upon presentation, a physical examination revealed no abnormalities on palpation, auscultation, and visual inspection. A routine plasma biochemistry panel (FUJIFILM DRI-CHEM 7000 V; FUJIFILM Co., Tokyo, Japan) revealed markedly increased levels of alanine aminotransferase (ALT), aspartate aminotransferase (AST), alkaline phosphatase (ALP), and gamma-glutamyl transferase (GGT) activities, and mildly increased levels of total bilirubin (T-BiL) and C-reactive protein (CRP) (ALT: 708 IU/L (reference interval (RI), 17–78 IU/L); AST: 170 IU/L (RI, 17–44 IU/L); ALP: 1752 IU/L (RI, 47–254 IU/L); GGT: 28 IU/L (RI, 5–14 IU/L); T-BiL: 2.2 mg/dL (RI, 0.1–0.6 mg/dL); and CRP: 2.2 mg/dL (RI, <0.7 mg/dL) (Table 1). Biochemical abnormalities were suggestive of hepatocellular injury and cholestasis [9]. Moreover, the increased CRP levels were suggestive of inflammation or neoplasia [10]. Coagulation tests were performed using citrated plasma (3.2%) at a commercial laboratory (FUJIFILM VET Systems, Tokyo, Japan). These tests revealed mildly increased fibrin degradation products (7.1 µg/mL; RI, <5 µg/mL) (Table 1). The findings of a complete blood count (ProCyte Dx Hematology Analyzer; IDEXX Laboratories, Tokyo, Japan), blood smear examination, and thoracic radiography were unremarkable. Abdominal radiography revealed moderate hepatomegaly and splenomegaly. Abdominal ultrasonographic findings were suggestive of cholecystitis, characterized by a moderately thickened gallbladder wall and multiple small hypoechoic splenic nodules. No abnormalities were observed in the other abdominal organs. The dog was administered ursodeoxycholic acid (10 mg/kg, PO, q12h) (URSO^®^; Mitsubishi Tanabe Pharma Corporation, Osaka, Japan) and a liver supplement containing S-adenosyl methionine and silymarin (three tablets/head, PO, q24h) (HEPAACT^®^PLUS; ZENOAQ Co., Ltd., Tokyo, Japan) from day 1 until day 21. On day 21, the dog underwent cholecystectomy for suspected extrahepatic bile duct obstruction, as well as a liver biopsy and splenectomy due to no improvement in ALT (849 IU/L), AST (159 IU/L), ALP (1898 IU/L), GGT (32 IU/L), and T-BiL levels (2.3 mg/dL). Prominent enlargement of the gallbladder was noted. The liver lobes showed a diffuse yellow color and moderate enlargement. The spleen was moderately enlarged without macroscopic nodules. The gallbladder was filled with small amounts of sandy choleliths. A bile culture performed in a commercial laboratory (Sanritsu Zelkova, Kanagawa, Japan) was negative. Wright–Giemsa-stained cytologic specimens obtained during surgery from the liver and spleen showed that moderate numbers of sGLs were present in the liver and spleen (Figure 1A,B). The nuclei were smaller than the cytoplasm of the neutrophils, up to approximately 1.5 times the size of the RBCs, round, occasionally indented, and paracentral to eccentric with coarsely granular chromatin. Multiple poorly distinct, round nucleoli were occasionally observed in the nuclei of the lymphocytes. A small to moderate amount of basophilic cytoplasms were present, often containing multiple small intracytoplasmic red-to-magenta granules with a relatively high N/C ratio. Moderate numbers of leukocytes (mainly neutrophils), clusters of well-differentiated hepatocytes, and marked canalicular plugging were observed in the liver, indicating cholestasis. Mild extramedullary hematopoiesis was also observed in the spleen.

**Figure 2:**
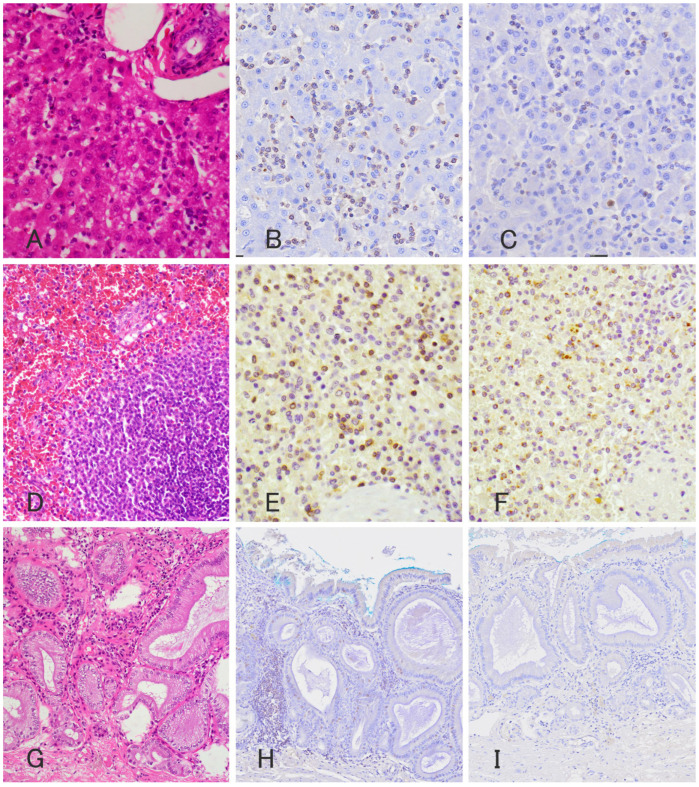
Photomicrograph of histopathology (**A**,**D**,**G**) and immunohistochemistry of anti CD3 antibody (**B**,**E**,**H**) and anti-granzyme B antibody (**C**,**F**,**I**) of the liver (**A**–**C**), spleen (**D**–**F**), and gallbladder (**G**–**I**). Histopathology revealed infiltration of moderate numbers of small lymphocytes with relatively spared hepatic lobular architecture. These neoplastic lymphocytes are stained positively for anti-CD3 antibody (**B**) and anti-granzyme B antibody (**C**) and negative for anti-CD20 antibody. Histopathology of the spleen showed diffuse congestion and enlarged red pulps with small lymphocytes. Approximately 50% of the lymphocytes are stained positively for anti-CD3 antibody (**E**) and anti-granzyme B antibody (**F**) and negative for anti-CD20 antibody. In the gallbladder, approximately 30% of infiltrating small lymphocytes are stained positively for anti-CD3 antibody (**H**), but unlike those in the spleen and liver, negative for anti-granzyme B antibody (**I**).

The spleen, gallbladder, and liver biopsy samples were subjected to histopathological evaluation and reviewed by a board-certified veterinary anatomic pathologist (Dipl. ACVP) (Figure 2). Hematoxylin-eosin (H and E) staining of multiple sections of the spleen revealed diffuse mild congestion, a few lymph follicles, an enlarged red pulp, mild-to-moderate extramedullary hematopoiesis, and a mixture of lymphocytes, macrophages, and blood components (Figure 2D). The lymph follicles were relatively small, consisting mainly of small lymphocytes equivalent in size to 1 to 1.5 erythrocytes, with less than one mitotic figure per high magnification field (0.237 m^2^). The destruction of tissue architecture in the spleen was not evident. H and E staining of multiple gallbladder sections revealed that the gallbladder mucosa was mildly hyperplastic, with mild infiltration of inflammatory cells, mainly small lymphocytes and plasma cells, in the lamina propria (Figure 2G). H and E staining of multiple liver sections revealed mild atrophy of the hepatic lobules with multifocal random loss of hepatocytes, infiltration of a moderate number of small lymphocytes and fewer neutrophils, and mild destruction of the hepatic lobular architecture (Figure 2A). Immunohistochemical staining was performed for CD3, CD20, and granzyme B using the following antibodies: a mouse anti-CD3 polyclonal antibody (Dako, rabbit polyclonal, Santa Clara, CA, USA, 1:200), rabbit anti-CD20 polyclonal antibody (Thermo Scientific, rabbit polyclonal, Cheshire, UK, 1:400), and rabbit anti-granzyme B polyclonal antibody (Abcam, rabbit polyclonal, Tokyo, Japan, 1:100) for the spleen, liver, and gallbladder. Hepatic immunohistochemistry revealed that most of the small lymphocytes were cytoplasmically positive for CD3 and granzyme B (Figure 2B,C) and negative for CD20. A splenic immunohistochemistry revealed that approximately 50% of the small lymphocytes scattered in the red pulp were positive for CD3 and granzyme B (Figure 2E,F). A gallbladder immunohistochemistry revealed that approximately 30% of the small lymphocytes in the lamina propria were positive for CD3 but negative for granzyme B (Figure 2H,I). A PCR for antigen receptor gene rearrangement (PARR) analysis was performed at the University of Tokyo following the method described previously [3] to examine the clonal rearrangement of immunoreceptor genes in liver cytology specimens. Monoclonal rearrangements of the T-cell receptor ɤ (TCRɤ) chain gene were detected, but no clonal patterns indicating immunoglobulin heavy chain (IgH) gene rearrangements were found. The diagnosis was atypical HSTCL predominately composed of sGLs concurrent with lymphocytic cholecystitis and mild gallbladder mucocele because a blood smear examination showed no lymphocytosis, no appearance of sGLs, and no neoplastic lymphocytes morphology suggestive of chronic lymphocytic leukemia–large granular lymphocytic leukemia (GL–CLL). Based on the mitotic figures, the HSTCL of this case is histologically low-grade [11].

**Figure 3:**
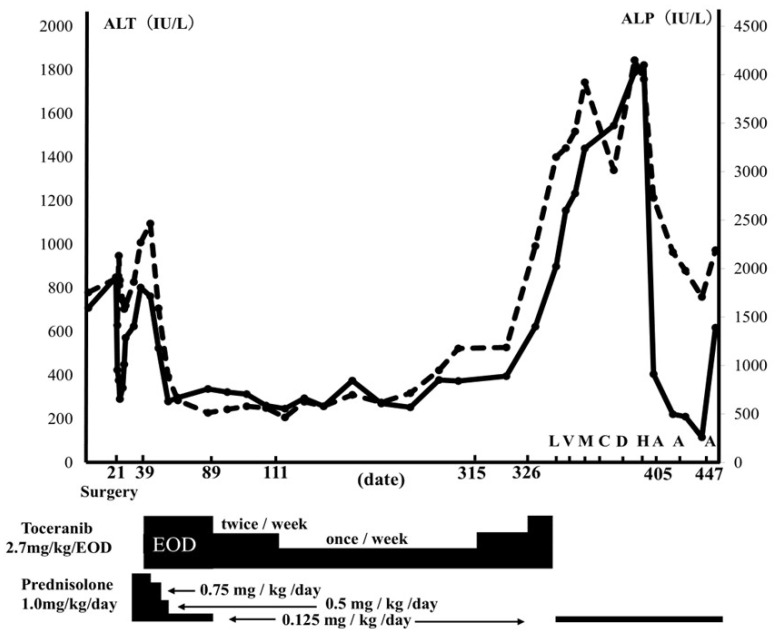
Clinical course of this case. The graph shows ALT (solid lines) and ALP (dotted lines) transition, and each drug’s dose transition and administration date. L: L-asparaginase, 400 U/kg, SC, on day 341. V: vinblastine, 2.0 mg/m^2^, IV, on day 348. M: Masitinib, 11.1 mg/kg/day, PO, from day 355 until day 361. C: carboplatin, 200 mg/m^2^, IV, on day 362. D: doxorubicin, 30 mg/m^2^, IV, on day 383. H: chlorambucil, 2 mg/head/each other day, PO, from day 398 until 404. A: nimustine, 25 mg/m^2^, IV, on day 405, 426 and 447.

After surgery, a chemotherapeutic agent sensitivity examination (CASE) [12,13,14] was performed at a commercial laboratory (Airdec mini, Tokyo, Japan) using liver biopsy tissue. Postoperatively, trepibutone (1 mg/kg, q12h) (SUPACAL; OHARA Pharmaceutical Co., Ltd., Shiga, Japan) was started. By day 22, T-BiL concentration (0.2 mg/dL) had normalized, and by day 25, CRP (0.3 mg/dL) had also normalized. However, starting from day 26, ALT and ALP levels began to rise (ALT; 341 IU/L, ALP; 1673 IU/L), prompting the initiation of prednisolone (1 mg/kg, q24h) (KYORIN Pharmaceutical, Tokyo, Japan). However, by day 39, ALT and ALP levels (ALT: 802 IU/L, ALP: 2267 IU/L) had further increased (Figure 3). On day 39, toceranib (2.7 mg/kg, per os (PO) every other day, administered four times a week) (Palladia^®^; Zoetis, Tokyo, Japan) as chemotherapy was started, based on the result of CASE (Table 2). We informed the owner in advance that toceranib has not been reported to be used for canine HSTCL so far, and thus, the effectiveness of toceranib is unclear for canine HSTCL. Subsequently, the owner agreed to use toceranib as a chemotherapeutic agent. ALT and ALP quickly decreased after the start of toceranib administration (Figure 3). Prednisolone was stopped on day 89. However, because of mild neutropenia (1.68 × 10^3^/µL, RI: 2.95–11.64 × 10^3^: grade 1 according to the Veterinary Cooperative Oncology Group-Common Terminology Criteria for Adverse Events) [15] observed with toceranib, the dose was tapered from four times a week to twice a week (2.7 mg/kg/day), and then further to once a week (2.7 mg/kg/day) (Figure 3). Although ALT and ALP levels were slightly elevated at the lowest dose (2.7 mg/kg/day once per week), no changes in clinical presentation were observed. An abdominal ultrasound revealed no changes in liver echogenicity, no appearance of mass lesions, and no signs of further organ invasion on day 305 compared to the initial presentation. The dog remained in good condition, and ALT and ALP levels were 395 IU/L and 1185 IU/L, respectively (Figure 3). However, on day 326, the dog exhibited abdominal distention, and abdominal ultrasonography revealed mild anechoic peritoneal effusion, and the ALT and ALP values were elevated to 622 IU/L and 2234 IU/L, respectively (Figure 3). The peritoneal effusion was characterized as a high-protein transudate (total protein: 4.5 g/dL) with a cell count of <5000/µL and low numbers of sGLs (1% of nucleated cells), which were morphologically similar to those noted in liver cytology during surgery. Due to concerns regarding inadequate toceranib dosage, the toceranib dosage was adjusted to 2.7 mg/kg every other day for 2 weeks despite potential adverse events associated with chemotherapy. However, by day 341, the peritoneal effusion had significantly increased, and liver enzymes had risen further (ALT: 898 IU/L, ALP: 3150 IU/L) (Figure 3). Then, prednisolone was resumed at 0.125 mg/kg/day starting on day 341. L-asparaginase (400 U/kg, subcutaneous injection) (Leunase; Kyowa Kirin, Tokyo, Japan) was administered on day 341, vinblastine (2.0 mg/m^2^, intravenously (IV)) (Exal; Nippon Kayaku Co., Tokyo, Japan) on day 348, masitinib (11.1 mg/kg, PO, q24h) (Masivet^®^; AB Science, Paris, France) from day 355 to day 361, carboplatin (200 mg/m^2^, IV) (CARBOPLATIN; Nichi-Iko Pharmaceutical Co., Ltd., Toyama, Japan) on day 362, doxorubicin (30 mg/m^2^, IV) (ADRIACIN; SANDOZ Japan Co., Ltd., Tokyo, Japan) on day 383, and chlorambucil (2 mg/head, every other day, PO) (LEUKERAN; Aspen Pharmacare Australia, st Leonards, NSW, Australia) from day 398 until day 404 (Figure 3). However, there was no improvement in the peritoneal effusion; liver enzymes continued to increase, and diarrhea developed as a clinical manifestation. On day 405, the dog developed severe ascites, with ALT and ALP levels reaching 1822 and 3954 IU/L, respectively. ACNU (25 mg/m^2^, IV) (Nidran; Daiichi-Sankyo, Tokyo, Japan) was started on day 405. By day 426, the peritoneal effusion had decreased from severe to mild, and liver enzymes had decreased (ALT: 221 IU/L, ALP: 2167 IU/L). Additionally, the diarrhea had completely resolved. Subsequently, ACNU was administered twice, on days 426 and 447. However, on day 457, liver enzymes re-elevated (ALT: 618 IU/L, ALP: 2188 IU/L), and ascite accumulation worsened. Additionally, moderate thrombocytopenia (platelet count: 99 × 10^3^/µL, RI: 148–484 × 10^3^/µL) and regenerative anemia (packed cell volume 25.4%, RI: 37.3–61.7%; reticulocytes 342.1 × 10^3^/µL, RI: 10–110 × 10^3^/µL) were newly diagnosed. On day 460, the patient developed respiratory distress and died. The owner declined an autopsy, and the cause of death was not determined.

## 3. Discussion

To the best of our knowledge, this is the first report of canine HSTCL exhibiting long-term survival using a combination of toceranib and prednisolone administration after splenectomy. Previous studies have consistently indicated an extremely poor prognosis for HSTCL [1,2,3,4,16,17,18,19]. Keller et al. reported that among nine cases of canine HSTCL, the survival time ranged from 1 to 196 days, with a median survival time of 4 days [2]. HSTCL is characterized as a T-cell lymphoma predominantly affecting the liver and spleen without involvement of peripheral lymph nodes. The lymphoma typically consists of intermediate-to-large-sized lymphocytes, with GLs containing azurophilic granules in the cytoplasm in many cases [2]. Unlike previous reports [2], the proliferating lymphocytes in this case were small in size. However, cytology stained with Wright–Giemsa confirmed sGLs, with positive findings of CD3 and granzyme B staining in immunohistochemistry and positive TCRɤ in the PARR analysis. These findings confirmed that the sGLs were neoplastic lymphocytes, indicating HSTCL with sGLs. In addition, this case of HSTCL of sGL is histologically graded as low based on the mitotic figures [11]; however, further studies would be needed to evaluate association of histologic grade and clinical behavior for each subtype of lymphoma [20]. The differential diagnosis of lymphoma, in this case, included aleukemic or subleukemic chronic lymphocytic leukemia (CLL) that might have spread from primary gastrointestinal lymphoma. A previous report described a dog with aleukemic GL–CLL [21], wherein asymptomatic neutropenia, thrombocytopenia, and lymphopenia persisted for 3 years. Bone marrow examination revealed 44% GLs, leading to a diagnosis of GL–CLL. However, in this case, aleukemic LGL–CLL was less likely to be the cause, as described in a previous report [21]. Due to the absence of increased or decreased lymphocytes, the absence of sGLs and neoplastic morphology of lymphocytes in the peripheral blood, and the absence of cytopenia, such as anemia, thrombocytopenia, and neutropenia. Other differential diagnoses included hepatic and splenic metastasis of gastrointestinal lymphoma involving GLs [16]. However, given the absence of typical clinical gastrointestinal signs that often accompany gastrointestinal lymphoma [22,23], as well as the lack of a primary lesion in the gastrointestinal tract observed during abdominal ultrasound examination, this possibility was considered unlikely. Therefore, the diagnosis of HSTCL was made for this dog.

In addition, a unique feature of this case is that the neoplastic GLs in HSTCL were smaller in size compared to what is typically described in existing literature [1,2,3,4,16,17,18,19]. The histopathological features observed with HE staining mimicked lymphocytic cholangiohepatitis, primarily due to the small size of the lymphocytes and the absence of significant tissue architecture destruction. Therefore, immunohistochemistry and PARR analysis were necessary to obtain a definitive diagnosis of canine HSTCL. Cytology using Wright–Giemsa staining is essential for the morphological diagnosis of HSTCL with GLs because the fine granules within the GLs are difficult to detect with H and E staining alone. In our case, the infiltration of small lymphocytes was challenging to distinguish from cholangiohepatitis, especially given the concurrent cholangitis with mild mucocele, which has been reported as a commonly associated disease [24,25]. Lymphocytic cholangiohepatitis is more commonly observed in cats, where significant lymphocytic infiltration can occur. In the present case, the degree of lymphocytic infiltration was milder than classic lymphocytic cholangiohepatitis observed in cats. This made diagnosing HSTCL based primarily on the findings from the HE sections more challenging. The neoplastic lymphocytes in previously reported HSTCL cases were usually mainly intermediate to large, and HSTCL with predominantly small-sized lymphocytes has not been reported. However, in our case, most lymphocytes contained small amounts of fine intracytoplasmic granules, which is an atypical finding for inflammation. This prompted us to conduct additional immunohistochemical and genetic testing. HSTCL with sGLs may have been underdiagnosed and should be considered as a differential diagnosis in similar cases.

Currently, no effective chemotherapeutic protocols are known to achieve long-term survival in canine HSTCL, and there are no reports on long-term prognosis. CHOP-based protocols, CCNU, and ACNU are often used as chemotherapeutic agents, but none have resulted in a favorable prognosis [2,26]. In this case, three possible factors may have influenced the long-term clinical outcome. First, the neoplastic lymphocytes responded to the CASE-based therapeutic agent, toceranib. Second, the neoplastic lymphocytes were more mature compared to those typically observed in previously reported HSTCL cases. Finally, surgical splenectomy contributed to the cytoreduction of the sGL lymphoma. Given the poor prognosis of HSTCL and the lack of an established effective chemotherapy protocol, we performed the CASE in this study. Based on the CASE results, we selected toceranib as the first-line therapeutic agent. Although there are few reports on the use of toceranib for the treatment of lymphoma in dogs, Yamazaki et al. [27] reported that the *c-kit* expression rate was higher in T-cells than in B-cells in a study of five dogs with multidrug-resistant lymphomas. Two of these five multidrug-resistant lymphomas showed partial response, suggesting that toceranib might have some effect in the treatment of lymphoma.

In the present report, we investigated six c-kit mutations, often investigated in mast cell tumor cases [5,6,7], using cytology specimens of day 21 after the death of the case. As a result, all *c-kit* mutation was not detected. However, we consider c-kit DNA sequencing necessary to detect the mutation, as well as further investigation. In this case, c-kit DNA sequencing was not possible. And, it was not possible to investigate for factors such as PDFFR and VEGFR, which are reported to be involved in tumor suppression by toceranib [5,6,7]. In our opinion, it is urgent to elucidate the mechanisms by which toceranib was effective with respect to all tumors, not just HSTCL, in the future. Liver enzyme levels in this dog were elevated despite treatment with prednisolone and hepatoprotective drugs, such as ursodeoxycholic acid and SAMe, and the dog also had a decreased appetite. However, toceranib lowered liver enzyme levels and improved appetite. Routine blood tests showed mild neutropenia; therefore, toceranib was administered at reduced doses as needed. Although the reduced dose of toceranib caused a slight increase in liver enzymes, it was effective until day 326 without clinical symptoms and without obvious mass lesions or enlarged intra-abdominal lymph nodes on ultrasound examination. The cause of death was undetermined; however, disseminated intravascular coagulation, immune-mediated hemolytic anemia, or hemophagocytic syndrome were considered based on clinicopathological data, which might be secondary to HSTCL. Toceranib was effective in treating HSTCL in this case, achieving a longer-term prognosis compared to the treatment in previous reports. These results suggest that toceranib should be considered a treatment option for HSTCL. Additionally, HSTCL with predominantly sGLs may have a better prognosis than that of predominantly large GLs. A recent report by Yale et al. [16], which consisted of 65 cases of canine LGL lymphoma (including 19 cases of HSTCL), classified the size of the neoplastic lymphocytes as large (>2 RBCs) or intermediate (1.5–2.0 times RBCs). They also revealed that lymphocyte size was significantly associated with long-term survival. In our case of lymphoma with sGLs (up to 1.5 RBCs), longer survival was observed, suggesting that the size of neoplastic lymphocytes may help predict outcomes. Although a histopathological grading system for lymphoma based on mitotic figures has been proposed, a grading scheme for each type of lymphoma has not been established. Previous reports failed to demonstrate a correlation between histologic grade and the clinical behavior of lymphoma with large or intermediate granular lymphocytes; therefore, evaluating cell size may be helpful. The small size of the tumor lymphocytes might have been a factor in achieving the long-term prognosis in the present case. Finally, the surgical removal of the spleen may have reduced the number of tumor cells, and postoperative therapy may have efficiently suppressed residual disease in the liver. There are few reports of HSTCL to date; however, in the report by Keller et al. [2], one out of the nine patients underwent surgery, specifically for cholecystectomy and liver biopsy, but none underwent splenectomy. In a report by Yale et al. [16] on 65 cases of LGL lymphoma, 19 cases of HSTCL were included. However, only two out of the 65 cases underwent surgery (one involved the removal of the affected lateral eye due to disseminated LGL lymphoma, and the other involved jejunal LGL lymphoma). Notably, these two cases did not include hepatosplenic lymphoma. In our case, the tumor cells were small, there were no serious clinical symptoms, and the lymphoma was restricted to the liver and spleen. Given the number of neoplastic lymphocytes in the liver and the aforementioned features, lymphoma with sGL was diagnosed at an early stage. Furthermore, the reduction in neoplastic cells by splenectomy may have also contributed to the long-term clinical outcome.

This study has some limitations. First, flow cytometry and one immunostaining were not performed in this case because these examinations are currently not available in Japan; since TCRγδ and CD11d expression is a characteristic of the HSCTCL immunophenotype, it is hoped that these examinations will become available in Japan in the future. Second, the primary lesion of the GL lymphoma in this case was not definitively determined; bone marrow examination and gastrointestinal endoscopy may be necessary to reliably exclude hepatosplenic infiltration from CLL or gastrointestinal lymphoma. Third, the clinical efficacy of toceranib in the treatment of HSTCL remains unclear. Although our case showed a good clinical outcome, the chemotherapeutic agent was selected based on CASE analysis for this particular neoplastic cell, and the size of the neoplastic cells in this case differed from previously reported HSTCLs [1,2,3,4,16,17,18,19]. Therefore, further studies are warranted to evaluate the efficacy of toceranib in the treatment of HSTCL.

In conclusion, this is the first report of canine HSTCL showing long-term survival following toceranib administration after a splenectomy. The clinicopathologic features of HSTCL with sGL may differ from those of previously reported HSTCL with intermediate to large GLs. Moreover, toceranib can be considered a treatment option for HSTCL. Further investigation with larger case numbers is warranted to evaluate the effectiveness of toceranib for canine HSTCL.

## Figures and Tables

**Table 1:** On day 1, the CBC, chemistry, and hemostatic test results. Reference intervals are from our laboratory. Arrow indicates within reference interval (RI) or not. ↑: above RI, -: within RI, ↓: under RI.

	Day 1	Unit	Reference Interval	Parameters		Day 1	Unit	Reference Interval	Parameters
RBC	6.22	×10^6^/μL	5.65–8.87	-	Total proteins	7.7	g/dL	5.0–7.8	-
PCV	44.8	%	37.3–61.7	-	Albumin	3.7	g/dL	2.6–4.0	-
Hemoglobin	14.2	g/dL	13.1–20.5	-	ALT	708	IU/L	17–78	↑
MCV	72	fL	61.6–73.5	-	AST	170	IU/L	17–44	↑
MCH	22.8	pg	21.2–25.9	-	ALP	1752	IU/L	47–254	↑
MCHC	31.7	g/dL	32.0–37.9	↓	GGT	28	IU/L	5.0–14	↑
Reticulocytes	26	×10^3^/µL	10–110	-	Total Bilirubin	2.2	mg/dL	0.1–0.8	↑
WBC	5.3	×10^3^/µL	5.05–16.76	-	Ammonia	20	µg/dL	16–78	-
Neutrophils	3.16	×10^3^/µL	2.95–11.64	-	Glucose	102	mg/dL	78–128	-
Lymphocytes	1.19	×10^3^/µL	1.05–5.1	-	Cholesterol	253	mg/dL	115–320	-
Monocytes	0.66	×10^3^/µL	0.16–1.12	-	Triglycerides	131	mg/dL	30–133	-
Eossinophils	0.24	×10^3^/µL	0.06–1.23	-	Urea	16.1	mg/dL	10.0–29.2	-
Basophils	0.9	×10^3^/µL	0–0.1	-	Creatinine	0.96	mg/dL	0.4–1.4	-
Platelets	166	×10^3^/μL	148–484	-	Phosphorous	3.6	mg/dL	1.9–5.0	-
PT	6	s	5.0–6.0	-	Calcium	10.7	mg/dL	9.3–12.1	-
APTT	12	s	11.0–16.0	-	C-reactive protein	2.2	mg/dL	<0.7	↑
Fibrinogen	369	mg/dL	128–420	-	Sodium	151	mmol/L	141.0–152.0	-
AT	150	%	114–179	-	Potassium	4.4	mmol/L	3.8–5.0	-
FDPs	7.1	µg/mL	0–5	↑	Chloride	102	mmol/L	102–117	-
D-dimer	1.6	µg/mL	0–2	-					
TAT	0.2	ng/mL	0–0.2	-					

PCV, packed-cell volume; MCV, mean cell volume; MCH, mean corpuscular hemoglobin; MCHC, mean corpuscular hemoglobin concentration; PT, prothrombin time; APTT, activated partial thromboplastin time; AT, anti-thrombin; FDPs, fibrin degradation products; TAT, thrombin antithrombin complex; ALT, alanine aminotransferase; AST, asparate aminotransferase; ALP, alkaline phosphatase; GGT, gamma glutamyl transferase.

**Table 2:** Results of chemoterapeutic agents sensitivity examination performed using liver biopsy tissue. Symbols indicate tumor cell growth inhibition rate of the drug. 〇: more than 50%, △: 20~50%, ×: less than 20%.

Chemoterapeutic Agent	Decision	Suppression Rate (%)
Toceranib	〇	92.70%
Carboplatin	〇	79%
Masitinib	〇	77.60%
Actinomycin D	〇	77.50%
Vinblastine	〇	71.50%
Doxorubicin	〇	63.80%
Cyclophosphamide	〇	60.80%
Methotrexate	〇	59.10%
Chlorambucil	〇	58.90%
Vincristine	〇	57.60%
Nimustine	〇	50.20%
Lomustine	△	37.90%
Prednisolone	×	17.40%

## Data Availability

All data underlying the results are available as part of the article, and no additional source data are required.
